# The impact of fitness app need support on women’s exercise adherence behavior: a serial mediation effect of self-efficacy and perceived health control

**DOI:** 10.3389/fpsyg.2026.1752995

**Published:** 2026-03-06

**Authors:** Wenlu Guan, Xiaocen Hao, Yanli Tang

**Affiliations:** Capital University of Physical Education and Sports, Beijing, China

**Keywords:** fitness app need support, perceived health control, self-efficacy, serial mediation effect, women’s exercise adherence behavior

## Abstract

**Background:**

In the global context of promoting active health, the issue of insufficient exercise adherence among women is increasingly prominent. As digital health intervention tools, the efficacy of fitness apps hinges on their ability to provide “need support” that satisfies users’ deep psychological needs. However, the internal psychological mechanism from need support to long-term behavioral adherence, particularly the serial mediation pathway for female users, requires further exploration.

**Methods:**

Grounded in Self-Determination Theory and Social Cognitive Theory, this study examined a sequential mediation model in which self-efficacy and health locus of control functioned as mediators. A cross-sectional questionnaire survey was administered to 721 female fitness app users recruited from 12 cities across 9 provinces in eastern, central, and western China. Data analyses were conducted using SPSS 27.0 and the PROCESS 4.0. Pearson correlation analyses and multiple linear regression were employed to test the associations and direct effects among variables, while mediation effects were examined using bootstrap procedures with 5,000 resamples and bias-corrected 95% confidence intervals. Indirect effects were considered statistically significant when the confidence intervals did not include zero.

**Results:**

(1) Fitness app need support significantly and positively predicted women’s exercise adherence behavior (total effect = 0.341, 95% CI [0.281, 0.400]). (2) Self-efficacy and perceived health control both played significant mediating roles, with mediation effect values of 0.098 and 0.079, respectively. (3) Self-efficacy and perceived health control formed a significant serial mediation pathway (effect value = 0.023). The total indirect effect, comprising this serial path and the two independent mediation paths, was 0.200, accounting for 58.65% of the total effect.

**Conclusion:**

Need support from fitness apps can directly promote women’s exercise adherence. It also indirectly fosters long-term behavioral persistence by sequentially enhancing users’ self-efficacy and perceived health control. This internal psychological sequence provides evidence of a chain mechanism for understanding the psychological “black box” of how digital health technologies influence behavior. The findings offer crucial theoretical and practical implications for the future design and optimization of fitness apps oriented toward women’s deep psychological needs.

## 1 Introduction

Globally, the concept of “active health” is gaining significant momentum. The World Health Organization has identified physical inactivity as the fourth leading risk factor for global mortality. Consequently, promoting regular physical activity among women has become a core issue in public health. As pillars of both family and society, women’s health status impacts not only individual well-being but also the health capital accumulation of families and society at large.

However, a “gender paradox” exists in this landscape. Although women generally show higher awareness of health information and stronger willingness to improve their health, they face numerous challenges in maintaining long-term exercise routines (O'Sullivan et al., 2025). Their participation rates and exercise adherence are often lower than men’s. They are also more prone to discontinuing due to social roles, body image anxiety, and negative experiences during exercise (Arigo et al., 2021). This phenomenon reveals that behind exercise behavior lie complex psychosocial mechanisms, extending beyond physiology and basic motivation, which require in-depth exploration. Importantly, the phenomenon under investigation is framed as gendered not merely because women have lower exercise adherence, but because the psychosocial mechanisms underlying exercise behavior are more salient and distinct for women than for men or other genders (Guo and Huang, 2025). The theoretical model adopted here focuses on the interplay of sociocultural pressures, gendered role expectations, and individual psychological factors, which directly address women’s unique barriers, barriers less pronounced or different for men (Morgan et al., 2024). Men face fewer such gender-specific constraints, and their exercise behavior is shaped by factors outside the model’s core focus. While other genders may have distinct exercise challenges, this study focuses on the well-documented gender paradox among cisgender women, and the model is tailored to unpack the gendered psychosocial mechanisms driving this discrepancy, making it more suited to women.

Against this backdrop, smartphone-based fitness apps are rapidly reshaping modern fitness ecosystems (Lisa et al., 2025). As key components of digital health technology, these apps are expected to solve adherence challenges. Their portability, interactivity, and personalization make them promising tools for providing sustained support, especially for women (Lyu et al., 2024). Existing research preliminarily confirms a link between fitness app use and initial motivation enhancement (Li, 2020).

Yet, why has the rapid growth in fitness app users not resulted in a corresponding improvement in long-term user retention and behavioral persistence (Liu et al., 2025)? This pattern suggests that merely possessing or using digital technology is insufficient to sustain health behaviors. Rather, the core issue lies in whether the content and functions of fitness applications adequately support users’ deep psychological needs, commonly referred to as need support. According to Self-Determination Theory, sustained behavior is driven by the satisfaction of three basic psychological needs: autonomy, competence, and relatedness (Ryan and Deci, 2020).

Recent empirical research applying Self-Determination Theory in digital health and physical activity contexts indicates that environments providing psychological need support are associated with higher autonomous motivation and more sustained engagement in health behaviors (Ntoumanis et al., 2021). However, despite the widespread availability of feature-rich fitness applications, academic research has not yet clearly explained how need support translates into long-term exercise adherence. This gap is particularly evident among female users, whose motivational and self-regulatory processes tend to be more sensitive to internal psychological factors. Accordingly, greater research attention should be directed toward users’ internal psychological processes rather than surface-level technological features.

Existing studies primarily examine direct associations between app features and behavioral intentions or rely on a single mediating variable, resulting in fragmented explanations. An integrated and sequential psychological pathway remains largely unexplored. In this regard, the present study proposes that self-efficacy and perceived health control function as two critical psychological mechanisms linking external need support to sustained exercise adherence. Self-efficacy, defined as individuals’ confidence in their ability to perform exercise behaviors and overcome obstacles, has consistently been identified as one of the most robust predictors in health behavior research (Zhang et al., 2025).

In summary, situated at the intersection of global digital health and positive psychology, this study moves beyond superficial descriptions of app utility. It aims to deeply analyze the internal psychological mechanism influencing exercise adherence among Chinese women. By proposing a serial mediation model involving self-efficacy and perceived health control, we seek to reveal the complete pathway from external technical support to internal psychological empowerment, and finally to sustained behavioral expression. This work will theoretically deepen the understanding of Self-Determination Theory in digital contexts and enrich research on mediation mechanisms in health behavior promotion. Practically, it will provide app developers with empirically-based strategies for woman-oriented design optimization. Ultimately, it aims to enhance health outcomes for women and help bridge the “gender adherence gap” in physical activity.

## 2 Literature review and research hypotheses

### 2.1 Fitness app need support and women’s exercise adherence behavior

Against the backdrop of rapidly developing digital health management, fitness apps serve as crucial tools for promoting physical activity. The mechanism through which the psychosocial support provided by their design influences user behavior adherence requires deeper investigation. To clarify the “App need support” construct, this section defines it explicitly, identifies corresponding fitness app features for Self-Determination Theory’s three psychological needs, and explains differential need satisfaction. App need support is a multi-dimensional construct referring to fitness apps’ functional design to meet users’ Autonomy, Competence and Relatedness needs, which promotes users’ fitness motivation and persistence (Xue et al., 2025). Fitness apps satisfy each psychological need through specific features. Competence need is met via clear feedback, goal setting and skill guidance, helping users perceive progress and enhance competence. Autonomy need is fulfilled by personalized program customization, flexible settings and optional modules, enabling users to initiate fitness behaviors independently. Relatedness need is addressed through social interaction features and professional guidance, fostering social connections and a sense of belonging. Fitness apps do not universally satisfy these three needs; satisfaction varies by app design and individual differences. Apps with integrated personalized, feedback and social features are more likely to meet all three needs, while single-function apps may only partially satisfy Competence need. Even with the same app, individual differences lead to varied perceived satisfaction (Adrián et al., 2024).

Self-Determination Theory posits that when individuals’ basic psychological needs are adequately met, their intrinsic motivation significantly increases, making long-term behavior adherence more likely (Daniel and Diego, 2025). According to this theory, fitness apps that effectively satisfy users’ needs for autonomy, competence, and relatedness can significantly enhance their participation motivation and behavioral persistence. Existing research indicates that a need-supportive environment within fitness apps can promote the adoption and maintenance of health behaviors by enhancing individuals’ psychological resources (Wang and Yoon, 2019). For instance, multiple empirical studies note that apps providing structured goal-setting and progress-tracking features enable users to perceive their improvements more clearly, thereby increasing the likelihood of adhering to exercise plans long-term (Brickwood et al., 2019).

From a need-support perspective, fitness apps can provide multi-dimensional support for female users. Regarding autonomy support, apps typically allow users to customize training goals and intensity based on their personal circumstances. This autonomy of choice can effectively enhance women’s sense of control and involvement (Seo et al., 2024). For competence support, features like visual data feedback, achievement badges, and progress notifications continuously reinforce women’s self-efficacy by allowing them to clearly perceive improvements in their physical fitness (Mazéas et al., 2025). Pertaining to relatedness support, built-in community features, social interactions, and coach guidance create positive social connections. These help alleviate the body anxiety and social evaluation pressure women might experience in traditional gym environments (Wang, 2023).

These support mechanisms collectively form a psychological support system that encourages women’s exercise participation. They provide richer psychological satisfaction during workouts, thereby strengthening intention and behavior for long-term adherence. This study therefore hypothesizes that a higher degree of need support from fitness apps leads to more positive and persistent exercise adherence behavior among female users. Consequently, the following hypothesis is proposed:

*H1*: Fitness app need support positively influences women’s exercise adherence behavior.

### 2.2 The mediating effect of self-efficacy

Self-efficacy consistently plays a vital role in health behavior promotion. According to Bandura’s Social Cognitive Theory, self-efficacy refers to an individual’s confidence in their ability to organize and execute courses of action required to achieve specific goals (Bandura, 2004). In the fitness context, it manifests as an individual’s belief in their capacity to overcome difficulties and adhere to regular exercise (Guo, 2022). Extensive empirical research has robustly confirmed that self-efficacy is a stable and powerful predictor of physical activity participation and adherence (Xu et al., 2026).

Compared to individuals with low self-efficacy, those confident in their ability to exercise tend to set more challenging fitness goals. They also demonstrate greater resilience against obstacles like fatigue, time conflicts, or waning motivation (Ryewon and Young-Ju, 2019). Consequently, they are more likely to develop long-term exercise habits.

Placing this theoretical lens within digital health interventions reveals a connection. The need support provided by fitness apps, theoretically grounded in Self-Determination Theory, aims to satisfy users’ basic psychological needs. Specifically, when an app uses progressive plans to foster a sense of mastery (“I can do this”), offers flexible choices to grant autonomy, and employs community encouragement to foster belonging, it creates positive, cumulative success experiences and feedback (Cotter et al., 2024). These experiences reinforce users’ confidence in their ability to perform exercise tasks, thereby enhancing their self-efficacy. For example, a study on mobile health applications found that regular progress tracking and positive feedback directly strengthened users’ belief in managing their health (Shibuta et al., 2023).

Furthermore, women may face unique barriers during exercise stemming from sociocultural factors, body image concerns, or safety perceptions. The inclusive, supportive environment of a fitness app, along with elements of “vicarious experience” and “social persuasion,” becomes particularly crucial. These features help female users build solid self-efficacy through observational learning and verbal encouragement. Therefore, the enhanced self-efficacy cultivated through app usage serves as internal motivation. This drives women to overcome difficulties and maintain long-term exercise behavior. Based on this reasoning, the following hypotheses are proposed:

*H2*: Fitness app need support positively influences women’s self-efficacy.

*H3*: Self-efficacy positively influences women’s exercise adherence behavior.

*H4*: Self-efficacy mediates the relationship between fitness app need support and women’s exercise adherence behavior.

### 2.3 The mediating effect of perceived health control

Perceived health control, defined as an individual’s inner belief in their ability to manage their health status and health behavior outcomes, is a key intrinsic motivational variable in health psychology for explaining health behavior adherence (Wallston et al., 1978). Within the context of digital health interventions, the range of need support functions provided by fitness apps serves to shape and strengthen this intrinsic sense of control in users. Existing research indicates that when individuals perceive that external environmental resources can effectively help them achieve health goals, they internalize this support, thereby enhancing their belief that “I can master my own health”, which reflects their perceived health control (Horvát et al., 2024).

Women’s exercise adherence often faces unique challenges at physiological, psychological, and sociocultural levels, making the role of perceived health control even more critical. When fitness apps accurately meet the personalized needs of female users during exercise through their functional design, users receive not only instrumental assistance but also experience an effective over their own fitness journey at cognitive and emotional levels (Wu et al., 2025). Thus, the enhancement of perceived health control is a key psychological bridge connecting external support to long-term adherence behavior. It transforms users from “passive participants” into “active managers,” making it more likely for short-term exercise behavior to become a stable lifestyle habit. Although direct research examining the relationships between fitness apps, perceived health control, and women’s exercise adherence is still limited, findings from offline contexts—such as health coaching and group support—indirectly corroborate the pathway whereby external support promotes sustained health behavior by enhancing the sense of control. Based on this, the following hypotheses are proposed:

*H5*: Fitness app need support positively influences women’s perceived health control.

*H6*: Perceived health control positively influences women’s exercise adherence behavior.

*H7*: Perceived health control mediates the relationship between fitness app need support and women’s exercise adherence behavior.

### 2.4 The serial mediating effect of self-efficacy and perceived health control

Focusing on the female user demographic and grounded in Self-Determination Theory and Social Cognitive Theory, this study attempts to construct a serial mediation model. This model elucidates the internal pathway through which self-efficacy and perceived health control link fitness app need support to exercise adherence behavior.

Fitness app need support can influence user behavior through multiple psychological mechanisms. According to Bandura’s Social Cognitive Theory, self-efficacy, defined as an individual’s belief in their capability to execute specific tasks, plays a central role in the process of health behavior change. Research finds that competence support provided by apps, such as personalized goal setting, progress feedback, and virtual rewards, can effectively enhance users’ exercise self-efficacy (Dieter et al., 2024). Increased self-efficacy not only directly predicts exercise adherence but may also further promote behavioral maintenance by influencing individuals’ cognitive assessments.

Perceived health control, a concept developed from the Health Belief Model, reflects an individual’s cognitive assessment of their control over health outcomes. Existing studies show that when individuals perceive a stronger control over health outcomes, they are more likely to adhere to health behaviors (Hagger et al., 2022). Fitness apps can strengthen users’ perceived health control by providing features like health data monitoring, scientific knowledge dissemination, and decision support.

**Figure 1:**
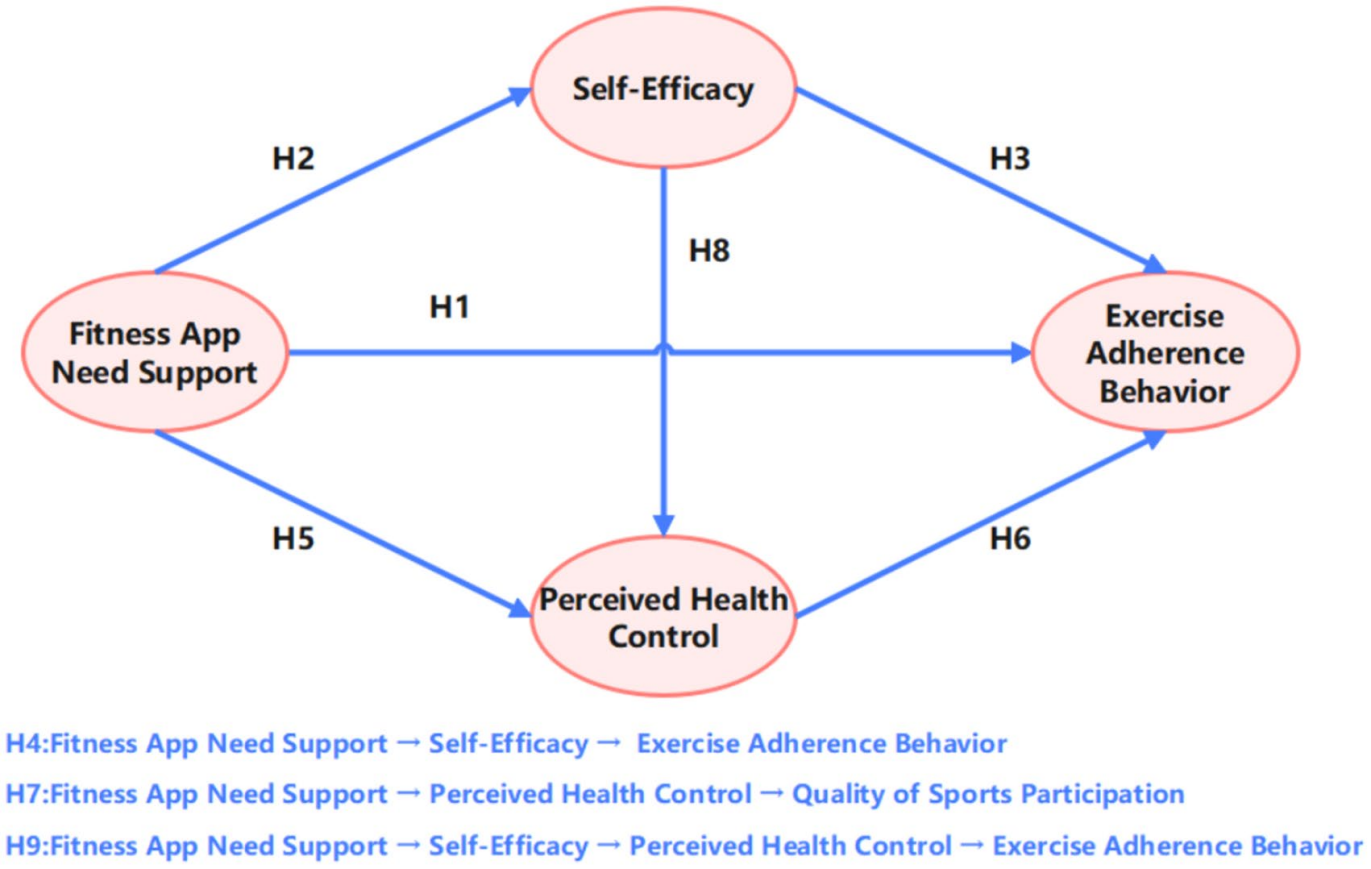
Hypothesized model.

Notably, an intrinsic relationship may exist between self-efficacy and perceived health control. Individuals with high self-efficacy are more inclined to adopt active strategies to manage their health, thereby enhancing their sense of control over health outcomes (Li et al., 2025). This sequential relationship suggests that the two variables may form a serial mediation pathway between need support and adherence behavior (Figure 1).

For the specific group of women, exercise behavior is more susceptible to psychosocial influences. Women often place greater emphasis on emotional experience and social support during exercise, while also being more prone to body image anxiety and fluctuations in self-efficacy. In view of this, the following research hypotheses are proposed:

*H8*: Self-efficacy positively influences perceived health control.

*H9*: Self-efficacy and perceived health control jointly serve as serial mediators in the relationship between fitness app need support and women’s exercise adherence behavior.

## 3 Research methods

### 3.1 Participants

This study investigated the impact of fitness app need support on women’s exercise adherence behavior, and the mediating roles of self-efficacy and perceived health control. The required sample size was estimated using G*Power 3.1 software. With parameters set to *α* = 0.05, statistical power (1-*β*) = 0.99, and a medium effect size (f^2^ = 0.15), the analysis indicated a minimum required sample size of 203.

The research adopted purposive sampling combined with snowball sampling, with strict criteria for participants who must have used fitness apps for at least 1 month, be over 18 years old, and come from different age and occupational groups. Fitness app users were identified through two-step screening, which included a preliminary question and a detailed inquiry about app usage. We selected nine provinces from the Eastern, Central, and Western regions of China. Two prefecture-level cities were randomly chosen from each province, totaling 12 cities. Data collection utilized both online and offline channels for random questionnaire distribution. A total of 400 paper-based questionnaires and 400 electronic questionnaires were distributed. Offline, researchers obtained the consent of venue management first and then contacted potential participants face-to-face in fitness-concentrated venues after explaining the research details, while online recruitment was conducted through fitness-related groups and platforms. Online questionnaires were distributed via Wenjuanxing after pre-survey optimization and with real-time validity checks, and offline paper questionnaires were distributed and collected on-site with immediate completeness verification and subsequent manual data entry, which effectively resolved the ambiguity in the original content.

After excluding 79 invalid responses (due to patterned responses, incomplete answers, or online completion times under 2 minutes), 721 valid questionnaires were retained, yielding a valid response rate of 90.13%. The final sample size meets the predetermined standard (Table 1).

**Table 1:** Distribution of demographic variables among participants.

Variable	Category	Frequency	Percentage (%)
Region	Eastern China	246	34.1
	Central China	256	35.5
	Western China	219	30.4
Age (years)	20 ~ 30	185	25.7
	31 ~ 40	183	25.4
	41 ~ 50	191	26.5
	51 ~ 60	162	22.5
Residence	Rural	354	49.1
	Urban	367	50.9
Education	Junior high or below	193	26.8
	High school	185	25.7
	Bachelor’s degree	161	22.3
	Postgraduate degree	182	25.2
Monthly income (RMB)	2,000 ~ 3,000	198	27.5
	3,000 ~ 4,000	180	25
	4,000 ~ 5,000	172	23.9
	More than 5,000	171	23.7

Geographically, participants were drawn from Eastern, Central, and Western regions, comprising 34.1, 35.5, and 30.4% of the sample, respectively. This indicates broad geographical coverage and relatively balanced distribution. Regarding age structure, all age groups were similarly represented: 20–30 years (25.7%), 31–40 years (25.4%), 41–50 years (26.5%), and 51–60 years (22.5%). This distribution ensures the inclusion of women across major age groups from youth to middle and older adulthood. This facilitates examination of age’s potential influence on exercise adherence.

In terms of urban–rural distribution, the sample showed nearly equal proportions of rural (49.1%) and urban (50.9%) respondents. This balanced structure provides a solid foundation for comparing differences in fitness resources, habits, and psychological mechanisms between urban and rural women.

Educational attainment within the sample displayed multi-level characteristics: junior high school or below (26.8%), high school (25.7%), bachelor’s degree (22.3%), and postgraduate degree (25.2%). The relatively continuous distribution across education levels enables analysis of cognitive differences in self-efficacy and perceived health control among women with different educational backgrounds.

Monthly income distribution was also relatively even across brackets: 2,000–3,000 RMB (27.5%), 3,000–4,000 RMB (25.0%), 4,000–5,000 RMB (23.9%), and above 5,000 RMB (23.7%). This gradient distribution reflects considerable economic heterogeneity in the sample. It supports exploration of income level’s potential influence on fitness app usage willingness and exercise adherence behavior.

Overall, the sample demonstrates good diversity and representativeness across key demographic variables including region, age, urban–rural residence, education, and income. This enhances the external validity of our findings. It also establishes a solid foundation for examining the serial mediation effect of self-efficacy and perceived health control between fitness app need support and women’s exercise adherence behavior.

### 3.2 Measurement instruments

#### 3.2.1 Fitness App Need Support Scale

The Need Support Scale in Fitness Apps, developed by Choi et al. (2015), was used. This scale was translated by Sun and Pan (2025) and validated within the Chinese context. It comprises three dimensions: Autonomy Support, Competence Support, and Relatedness Support. Each dimension contains 4 items, resulting in a total of 12 items. Responses are recorded on a 7-point Likert scale, ranging from 1 (“Strongly Disagree”) to 7 (“Strongly Agree”). The total score ranges from 12 to 84, with higher scores indicating greater perceived need support from the fitness app. In this study, the scale demonstrated good structural validity (KMO = 0.926; Bartlett’s test χ^2^ = 1993.890, *p* < 0.001) and good reliability (Cronbach’s *α* = 0.846).

#### 3.2.2 Exercise Adherence Scale

The Exercise Adherence Scale revised by Wang (2020), based on the original scale developed by Wang Shen, was adopted. This scale contains 15 items across three dimensions: Exercise Behavior, Effort Investment, and Emotional Experience. Responses are measured on a 5-point Likert scale, from 1 (“Not True”) to 5 (“Very True”). The scores for each dimension and the total scale are summed, with higher total scores indicating stronger exercise adherence. In this study, the scale showed good structural validity (KMO = 0.945; Bartlett’s test χ^2^ = 2594.556, *p* < 0.001) and good reliability (Cronbach’s *α* = 0.869).

#### 3.2.3 Self-Efficacy Scale

Self-efficacy was assessed using the General Self-Efficacy Scale (Luo and Xie, 2023). This scale includes 10 items. Responses are rated on a 4-point scale from 1 (“Not at all True”) to 4 (“Exactly True”). The total score ranges from 10 to 40, with higher scores indicating stronger self-efficacy. In this study, the scale exhibited good structural validity (KMO = 0.888; Bartlett’s test χ^2^ = 1089.646, *p* < 0.001) and acceptable reliability (Cronbach’s *α* = 0.772).

#### 3.2.4 Perceived Health Control Scale

The Multidimensional Health Locus of Control (MHLC) Scale, developed by Wallston et al. (1978), was used to measure perceived health control. This scale consists of 18 items across three dimensions: Internal, Powerful Others, and Chance. Each dimension contains 6 items. Responses are given on a 5-point Likert scale. Dimension scores range from 6 to 30, and the total score ranges from 6 to 90. Higher scores indicate a stronger tendency toward the respective control dimension. In this study, the scale demonstrated good structural validity (KMO = 0.936; Bartlett’s test χ^2^ = 2346.644, *p* < 0.001) and good reliability (Cronbach’s *α* = 0.850).

#### 3.2.5 Psychometric and methodological rationale

In consideration of the deliberate use of distinct Likert scales for different variables within this study, the rationale is grounded in the fundamental principle of psychometric appropriateness and the specific operational demands of each construct. The selection of scale length and anchoring descriptors was not arbitrary but derived from a thorough review of the established measurement literature pertaining to each variable (Imam et al., 2022). Key constructs within the research model possess differing conceptual densities and expected response distributions. Variables requiring respondents to make nuanced distinctions along a continuum of agreement or intensity were measured with a 7-point scale, as its greater granularity is better suited to capture variance in such contexts and aligns with common practice in related sub-fields (Ferrando et al., 2025). Conversely, variables assessing more concrete or behaviorally anchored frequencies were operationalized using a 5-point scale, a format widely validated for its clarity and reduced cognitive burden when evaluating overt behavioral estimates (Laura et al., 2022). This tailored approach ensures that each measurement instrument is optimized for its specific task: it enhances discriminant validity by preventing artificial correlations that can arise from uniform scale formats, respects the inherent properties of the constructs as defined in prior theoretical work, and improves response quality by matching the cognitive demands of the question to a psychometrically suitable response framework. Consequently, the variation in Likert scales constitutes a methodologically sound decision aimed at increasing the precision, validity, and overall rigor of the empirical data collected for this investigation.

### 3.3 Data processing and analysis

Data were analyzed using SPSS 27.0 and PROCESS 4.0. The main reason is that this study mainly focuses on testing the theoretically-driven moderation and serial mediation effects of the observed variables measured based on the established Likert scale. PROCESS employs a simple regression method and uses the bootstrap method to estimate the indirect effects, which is highly suitable for the analysis goals of this study (Zhao and Kou, 2024). In contrast, SEM is more suitable for modeling latent variables and evaluating the overall model fit, which is beyond the scope of this study. The procedures included: (1) Classifying, converting, and computing relevant valid data; (2) Testing for common method bias using Harman’s single-factor test; (3) Assessing the reliability (via Cronbach’s *α*) and structural validity (via Bartlett’s test) of the measurement tools; (4) Employing correlation analysis and linear regression to examine relationships between variables and to test the significant effects of body image, self-efficacy, and exercise motivation on women’s exercise adherence; (5) Using the Bootstrap method to test the simple mediation and serial mediation effects of exercise technology product usage intensity, perceived health control, and self-efficacy on middle-aged health anxiety.

## 4 Research results

### 4.1 Common method bias test

Common method bias was assessed using statistical procedures. An exploratory factor analysis (EFA) employing Harman’s single-factor test was conducted on all measurement items. The results showed that the first unrotated principal component accounted for 20.128% of the variance, which is below the critical threshold of 40%. This indicates that common method bias is not a severe concern in this dataset.

Although a single factor did not explain most of the variance, additional measures were implemented during the questionnaire design phase to control potential bias and further ensure robustness. These measures included: (1) separating the item order for independent and dependent variables; (2) utilizing scales from diverse sources and ensuring clear item wording; (3) employing anonymous responses. Subsequent analyses could also employ methods like controlling for an unmeasured latent methods factor or introducing a marker variable for supplementary verification. In summary, the data quality meets the basic requirements for statistical analysis.

### 4.2 Correlation analysis of research variables

Table 2 presents the descriptive statistics and correlation analysis for the core variables. The descriptive statistics show the central tendency and dispersion for each variable among the sample: Fitness App Need Support (Mean = 5.340, SD = 0.669), Exercise Adherence Behavior (Mean = 3.565, SD = 0.581), Self-Efficacy (Mean = 2.915, SD = 0.579), and Perceived Health Control (Mean = 3.217, SD = 0.603).

**Table 2:** Correlation analysis.

Variable	*M*	SD	Fitness app need support	Exercise adherence behavior	Self-efficacy	Perceived health control
Fitness app need support	5.340	0.669	1			
Exercise adherence behavior	3.565	0.581	0.380**	1		
Self-efficacy	2.915	0.579	0.249**	0.605**	1	
Perceived health control	3.217	0.603	0.462**	0.545**	0.501**	1

^*^p < 0.05; ^**^p < 0.01; ^***^p < 0.001, the same below.

The correlation analysis revealed significant positive correlations between Fitness App Need Support and Exercise Adherence Behavior (*r* = 0.380, *p* < 0.01). Need Support was also positively correlated with Self-Efficacy (*r* = 0.249, *p* < 0.01) and Perceived Health Control (*r* = 0.462, *p* < 0.01). Furthermore, Exercise Adherence Behavior showed relatively strong positive correlations with both Self-Efficacy (*r* = 0.605, *p* < 0.01) and Perceived Health Control (*r* = 0.545, *p* < 0.01). A significant positive correlation was also found between Self-Efficacy and Perceived Health Control (*r* = 0.501, *p* < 0.01). This pattern of significant correlations provides preliminary statistical support for further investigating the serial mediating role of self-efficacy and perceived health control in the relationship between fitness app need support and women’s exercise adherence behavior.

Table 3 presents the results of the multiple linear regression analysis conducted to examine the direct effects of fitness app need support, self-efficacy, and perceived health control on women’s exercise adherence behavior. The regression model provides various statistical metrics, including the unstandardized regression coefficient (B), standard error (SE), standardized regression coefficient (*β*), *t*-value, 95% confidence interval lower limit (LLCI) and upper limit (ULCI), Tolerance, and Variance Inflation Factor (VIF). These metrics collectively offer a comprehensive assessment of the relationship strength, significance, and model robustness.

**Table 3:** Results of multiple linear regression analysis.

Variable	B	SE	*β*	*t*	LLCL	ULCL	Tolerance	VIF
Constant	0.765	0.140		5.466	0.490	1.040		
Fitness app need support	0.135	0.027	0.154	4.974	0.082	0.189	0.786	1.273
Self-efficacy	0.442	0.032	0.44	13.928	0.379	0.504	0.749	1.335
Perceived health control	0.244	0.033	0.253	7.337	0.179	0.309	0.628	1.593

The regression coefficient for the constant term was 0.765 (SE = 0.140), with a *t*-value of 5.466, reaching statistical significance. Its 95% confidence interval was [0.490, 1.040]. This indicates a baseline prediction for women’s exercise adherence behavior significantly above zero when all independent variables are zero.

Regarding the independent variables, fitness app need support showed a regression coefficient of 0.135 (SE = 0.027), a standardized coefficient (*β*) of 0.154, and a *t*-value of 4.974. Its 95% confidence interval was [0.082, 0.189]. This demonstrates that need support significantly and positively predicts women’s exercise adherence behavior. Adherence behavior increases as the level of need support rises.

Self-efficacy yielded a regression coefficient of 0.442 (SE = 0.032), a standardized coefficient (*β*) of 0.440, and a *t*-value of 13.928. Its 95% confidence interval was [0.379, 0.504]. This indicates that self-efficacy has a more positive impact on compliance behavior. Its effect makes it the strongest predictor variable influencing compliance behavior in this model.

Perceived health control had a regression coefficient of 0.244 (SE = 0.033), a standardized coefficient (*β*) of 0.253, and a *t*-value of 7.337. Its 95% confidence interval was [0.179, 0.309]. This also shows a significant positive predictive effect on exercise adherence behavior.

Multicollinearity diagnostics for the model showed that all Tolerance values were above 0.6 (0.786 for Need Support, 0.749 for Self-Efficacy, 0.628 for Perceived Health Control). All VIF values were below 2 (specifically 1.273, 1.335, and 1.593), well under the critical threshold of 10. This indicates no severe multicollinearity issues among the independent variables. The stability and reliability of the regression results are thus assured.

In summary, this multiple linear regression model effectively reveals the independent influences of fitness app need support, self-efficacy, and perceived health control on women’s exercise adherence behavior. It provides an important statistical foundation for the subsequent analysis of the serial mediation effect.

### 4.3 Mediation effect test

Table 4 presents the detailed results of the mediation pathway analysis. The analysis indicates that the total effect of fitness app need support on women’s exercise adherence behavior is 0.341 (SE = 0.030). The bootstrap confidence interval for this total effect is [0.281, 0.400], confirming its statistical significance.

**Table 4:** Results of the mediation pathway analysis.

Effect	Path	Effect size	Standard error	LLCL	ULCL	Percentage of total effect
Total effect	Direct path	0.341	0.030	0.281	0.400	100.00
Direct effect		0.141	0.027	0.088	0.194	41.35
Total indirect effect		0.200	0.043	0.068	0.129	58.65
Indirect effect	Pathway 1	0.098	0.015	0.068	0.129	28.74
	Pathway 2	0.079	0.012	0.057	0.104	23.17
	Pathway 3	0.023	0.005	0.015	0.032	6.74

Further decomposition of this effect reveals a direct effect of 0.141 (SE = 0.027), with a confidence interval of [0.088, 0.194]. This direct effect accounts for 41.35% of the total effect, demonstrating a significant direct promoting influence of need support on adherence behavior.

Simultaneously, the total indirect effect, representing the combined mediation, is 0.200 (SE = 0.043). Its confidence interval is [0.116, 0.284], and it accounts for 58.65% of the total effect. This substantial proportion highlights the important collective role played by self-efficacy and perceived health control in the underlying mediation mechanism.

Regarding the Specific Mediation Pathways: The effect value for Path 1 is 0.098 (SE = 0.015), with a confidence interval of [0.068, 0.129], contributing 28.74% to the total effect. The effect value for Path 2 is 0.079 (SE = 0.012), with a confidence interval of [0.057, 0.104], contributing 23.17%. The effect value for Path 3 is 0.023 (SE = 0.005), with a confidence interval of [0.015, 0.032], contributing 6.74%. The confidence intervals for all three paths do not include zero, indicating that the mediation effects for each path are statistically significant.

**Figure 2:**
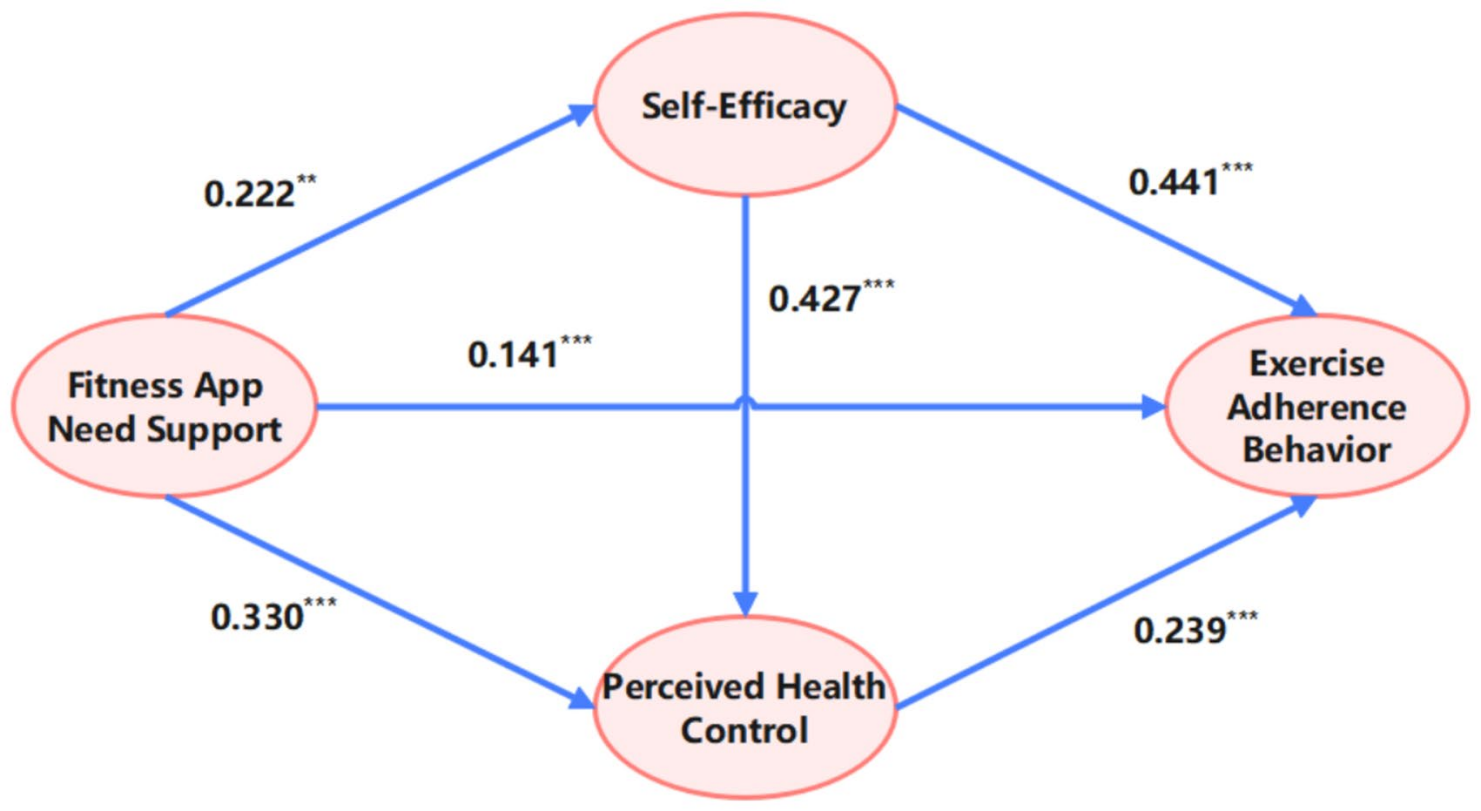
The chain mediation model of fitness app need support and women’s exercise adherence behavior.

In summary, self-efficacy and perceived health control form an explanatory serial mediation mechanism between fitness app need support and women’s exercise adherence behavior. The contributions of Path 1 and Path 2 are particularly notable. This further validates the critical role of the psychological mechanism in the behavioral adherence process (Figure 2).

## 5 Discussion

### 5.1 Explanation of the serial mediation mechanism and theoretical construction

The validated serial mediation model suggests a potential internal relationship between fitness app need support and women’s exercise adherence behavior. This relationship may operate through sequentially enhancing self-efficacy and perceived health control, forming a logically relevant potential psychological transformation pathway.

From a theoretical construction perspective, this potential chain relationship may be based on an integrated framework of Self-Determination Theory and Social Cognitive Theory. It preliminarily illustrates how external technological resources, through the sequential activation of internal psychological drivers, may potentially be translated into relevant behavioral performance (Xiao et al., 2025).

Specifically, the need support provided by fitness apps may act as a potential external enabling mechanism. It may first act upon an individual’s belief in their ability to execute behaviors, namely self-efficacy. When apps use features like progressive task design, immediate feedback, and social recognition to help users accumulate successful “I can do it” experiences during participation, their confidence in their ability to adhere to exercise may be strengthened (Cotter et al., 2024). This may constitute the starting point of the potential psychological transformation process.

Furthermore, the enhancement of self-efficacy may provide a potential cognitive and affective foundation for the formation of perceived health control (Zhu et al., 2025). According to Social Cognitive Theory, an individual’s belief in their behavioral control capability may influence their expectations regarding outcome control. When female users possess high self-efficacy regarding exercise behavior, they may be more inclined to attribute health outcomes to their own manageable behavioral factors, rather than attributing outcomes to external chance or powerful others. Consequently, they may gradually develop the internal belief that “I can master my own health,” namely perceived health control.

This cognitive transition from “behavioral efficacy” to “outcome control” may mark a potential psychological shift from passive participation to active management, and may also serve as a potential key cognitive hub for maintaining relevant adherence behavior. Thus, self-efficacy and perceived health control may form a mediating chain between need support and behavioral adherence potentially associated with temporal relevance and logical consistency. This path not only preliminarily suggests a multi-stage, dynamic potential psychological process through which external support may influence behavior, but also preliminarily highlights potential “cumulative reinforcement” and “sequential transmission” effects that may exist among psychological variables within digital health interventions.

### 5.2 Implications for fitness app design and optimization

Based on the discovered serial mediation mechanism, specific implications for fitness app design and optimization may be derived. The identified roles of self-efficacy and perceived health control suggest that fitness apps are not merely instrumental tools. They may function as psychological intervention platforms that potentially stimulate users’ intrinsic motivation and beliefs. Accordingly, app design may transcend the superficial logic of feature accumulation. It may shift toward a systematic construction centered on users’ deep psychological needs (Dewan et al., 2025). Particular attention may be paid to scientifically fostering users’ self-efficacy and perceived health control through content design and interactive mechanisms to potentially promote long-term behavior maintenance.

Regarding specific design strategies, apps may strengthen the competence support dimension. Implementing phased, visual goal-setting and feedback systems may help users accumulate successful experiences by completing “achievable challenges.” This process may enhance their confidence in their exercise capabilities. The system may provide specific, positive behavioral feedback promptly. This may allow users to perceive their progress clearly, potentially fostering a cognitive expectation of “I can do it.”

Furthermore, integrating mechanisms for observational learning and social persuasion may be relevant. For instance, featuring experience sharing from peers or users with similar health backgrounds within the community may provide vicarious reinforcement. This may further reinforce users’ self-efficacy beliefs.

Additionally, apps may focus on building a dual-path approach to cultivate perceived health control, combining autonomy support and informational support. For autonomy, grant users more control over the program and choices. Examples may include allowing customization of training plans, adjustment of difficulty parameters, and selection of preferred coaches. These features may enable users to experience a sense of active control over their health behaviors.

For informational support, the app may provide scientific and transparent interpretation of health data and knowledge. This may help users understand the potential link between their behaviors and health outcomes. It may guide them toward an internal attribution tendency, believing “health is controllable.” For example, pairing physiological indicator changes with behavioral suggestions may help users recognize the concrete benefits of regular exercise. These potential benefits may include mood regulation and improved physical capacity, thereby potentially strengthening their perceived control over health outcomes.

### 5.3 Implications for women’s fitness services and health promotion

The findings clearly indicate that fitness apps act as psychosocial resources. They stimulate and maintain intrinsic psychological drivers by satisfying users’ needs for autonomy, competence, and relatedness. This ultimately promotes long-term behavioral adherence. Specifically, fitness apps and related offline services should focus on creating a supportive interactive environment. Beyond providing training plans and data feedback, this environment should enhance users’ sense of participation and control (Aaya et al., 2024).

For instance, features like personalized goal negotiation, progress visualization, and positive feedback mechanisms allow users to perceive their ability improvements with each interaction. This helps them gradually build the internal belief that “health can be directed by their own actions” (Shibuta et al., 2023).

Moreover, the results suggest that health promotion policies and practices should emphasize the connection between psychological empowerment and behavioral maintenance. Many current health intervention programs still focus on knowledge dissemination or short-term behavior initiation. They often overlook the psychological transformation process from “knowing” to “doing” to “sustaining doing.”

Therefore, when formulating health promotion plans for women, the serial mediation model from this study can be instructive. Enhancing self-efficacy and perceived health control should be core intervention targets. These targets can be integrated into digital health management platforms and community health services.

Finally, this study also calls on fitness technology product and service providers to pay attention to women’s physiological and psychological changes across different life stages. Developing stage-appropriate support content is essential. For example, addressing health anxieties and body image issues potentially faced by middle-aged women, apps could integrate mental health guidance, functional training, and health data tracking. This holistic approach can help them maintain positive health identity and behavioral engagement while navigating age-related challenges.

In summary, the key pathway for enhancing the quality and effectiveness of future women’s fitness services and health promotion lies in transitioning from “technical support” to “psychological empowerment,” and upgrading from “single-behavior interventions” to “integrated psychological-behavioral support systems.”

## 6 Conclusions and prospects

### 6.1 Research conclusions

Grounded in Self-Determination Theory and Social Cognitive Theory, this study developed a theoretical model in which self-efficacy and perceived health control function as serial mediators. The study systematically examined the psychological mechanisms through which fitness app need support influences women’s exercise adherence behavior. Based on questionnaire data collected from 721 female fitness app users in eastern, central, and western regions of China, the main conclusions are as follows.

1. Fitness app need support has a significant positive influence on women’s exercise adherence behavior. This finding suggests that app features designed to support users’ basic psychological needs serve as important external resources for fostering long-term exercise adherence among women.
2. Self-efficacy and perceived health control each play significant mediating roles in the relationship between fitness app need support and women’s exercise adherence behavior. This indicates that fitness apps promote behavioral persistence indirectly by strengthening women’s confidence in their ability to engage in exercise and by enhancing their perceived control over health outcomes.
3. Self-efficacy and perceived health control together constitute a significant serial mediation pathway. Specifically, fitness app need support first enhances users’ self-efficacy, which subsequently strengthens their perceived health control, ultimately contributing to the maintenance of long-term exercise adherence.

### 6.2 Research limitations

Although this study systematically revealed the internal mechanism through a serial mediation model, contributing to theory and practice, several limitations remain for future research to address:

1. The cross-sectional design limits the strength of causal inference.
2. The sample’s representativeness and generalizability require cautious interpretation.
3. Reliance on self-reported data for variable measurement carries a risk of common method bias.
4. The model did not include other potential mediating or moderating variables.
5. The cultural universality of the findings needs verification through cross-cultural comparisons.
6. The investigation into the basic information of participants’ app usage was not sufficiently thorough. We failed to explicitly collect and analyze such core information as the specific types of apps actually used by participants and their usage time points, nor did it effectively control participants’ usage of different applications, leading to potential interference from confounding variables. In addition, this study failed to distinguish the differences in the effects between the inherent functions of the apps themselves and the demand support provided by the apps.

### 6.3 Future prospects

Building on the preliminary revelation of the serial mediation mechanism, future research could expand and deepen in the following aspects:

1. Enriching the theoretical model and contextual expansion. Future studies could introduce more diverse psychological and social variables (e.g., body image, social support, degree of motivation internalization) to examine their moderating or additional mediating roles within the serial pathway. This would build a more integrated theoretical framework. Simultaneously, testing this model across different cultural contexts or health behavior scenarios would enhance its universality and clarify boundary conditions.
2. Methodological longitudinal deepening and data diversification. While this cross-sectional study revealed associations and mediation pathways, causal inference is limited. Future work should employ longitudinal tracking or experimental intervention studies to dynamically capture the evolution of self-efficacy and perceived health control during app use. This would more accurately reveal the time-varying psychological mechanism. Combining objective behavioral data with subjective reports would also improve ecological validity and reliability.
3. Precision and personalization in fitness app design and health services. Future app development can leverage the identified serial pathway to design content modules with “psychological scaffolding” support. Examples include “progressive challenge tasks” for users with low self-efficacy or “health attribution guidance feedback” for those with weak perceived health control. Furthermore, exploring AI-based personalized recommendation systems could dynamically adjust support strategies based on users’ real-time psychological states and behavioral data, enabling a shift from a “one-size-fits-all” to a “tailored” digital transformation.
4. Introducing audience segmentation and a life-course perspective. This study focused on women broadly. Future research should segment female sub-groups by age, occupation, health status, or exercise stage. This would explore their differentiated psychological needs and behavioral responses when using fitness apps. Support mechanisms might follow unique pathways during pivotal life stages like pregnancy, postpartum, and menopause. Investigating these can inform more stage-appropriate health promotion strategies.
5. Promoting industry-academia collaboration and synergistic policy support. Strengthening dialog among academia, the health industry, and public policy is essential. Translating empirical evidence, like the serial mediation mechanism, into actionable product standards, service guidelines, or health promotion programs is crucial. This will upgrade fitness apps from “tools” to “empowerment vehicles,” ultimately building a sustainable ecosystem for promoting women’s health behaviors via digital technology.

## Data Availability

The original contributions presented in the study are included in the article/supplementary material, further inquiries can be directed to the corresponding author.
